# Linking epigenetic function to electrostatics: The DNMT2 structural model example

**DOI:** 10.1371/journal.pone.0178643

**Published:** 2017-06-02

**Authors:** Gilberto Cavalheiro Vieira, Gustavo Fioravanti Vieira, Marialva Sinigaglia, Vera Lúcia da Silva Valente

**Affiliations:** 1 Programa de Pós Graduação em Genética e Biologia Molecular, Universidade Federal do Rio Grande do Sul (UFRGS), Porto Alegre, RS, Brazil; 2 Departamento de Genética, Instituto de Biociências, Universidade Federal do Rio Grande do Sul (UFRGS), Porto Alegre, RS, Brazil; 3 Núcleo de Bioinformática do Laboratório de Imunogenética, Departamento de Genética, Universidade Federal do Rio Grande do Sul, Porto Alegre, RS, Brazil; 4 Instituto do Câncer Infantil, Porto Alegre, RS, Brazil; 5 Programa de Pós Graduação em Biologia Animal, Universidade Federal do Rio Grande do Sul (UFRGS), Porto Alegre, RS, Brazil; Universita degli Studi di Roma La Sapienza, ITALY

## Abstract

The amino acid sequence of DNMT2 is very similar to the catalytic domains of bacterial and eukaryotic proteins. However, there is great variability in the region of recognition of the target sequence. While bacterial DNMT2 acts as a DNA methyltransferase, previous studies have indicated low DNA methylation activity in eukaryotic DNMT2, with preference by tRNA methylation. Drosophilids are known as DNMT2-only species and the DNA methylation phenomenon is a not elucidated case yet, as well as the ontogenetic and physiologic importance of DNMT2 for this species group. In addition, more recently study showed that methylation in the genome in *Drosophila melanogaster* is independent in relation to DNMT2. Despite these findings, Drosophilidae family has more than 4,200 species with great ecological diversity and historical evolution, thus we, therefore, aimed to examine the drosophilids DNMT2 in order to verify its conservation at the physicochemical and structural levels in a functional context. We examined the twenty-six DNMT2 models generated by molecular modelling and five crystallographic structures deposited in the Protein Data Bank (PDB) using different approaches. Our results showed that despite sequence and structural similarity between species close related, we found outstanding differences when they are analyzed in the context of surface distribution of electrostatic properties. The differences found in the electrostatic potentials may be linked with different affinities and processivity of DNMT2 for its different substrates (DNA, RNA or tRNA) and even for interactions with other proteins involved in the epigenetic mechanisms.

## Introduction

Cytosine methylation plays a critical role in the regulation of gene expression in higher eukaryotes. Cytosine methylation is performed by DNA methyltransferases (DNMTs), which are classified into three subfamilies: DNMT1, DNMT2 and DNMT3a and 3b. DNMT2 is the smallest eukaryotic methyltransferase (approximately 400 amino acids) and the most widely distributed in animals, fungi, protists, and plants [1]. In agreement with structural conservation, different methods in various systems have shown that DNMT2 has DNA methyltransferase activity [2–5]. DNMT2 was first identified in mice and humans and is likely conserved among eukaryotes [6,7]. This enzyme is the only DNMT found in dipterans, including *Drosophila* [8].

Nevertheless, according to analyses of human and *Entamoeba* enzymes, its catalytic activity on DNA is very weak, suggesting that in some species have an alternative role [2,4]. For example, studies have associated DNMT2 with RNA interference in *Dictyostelium* and covalent histone modification in *Drosophila* [9,10]. Additionally, several studies have shown the occurrence of DNA methylation in *Drosophila* species [11–13]. Recent results reported showed methylation in the genome in *Drosophila melanogaster*, even in deficient-DNMT2 strains, indicating a probable independence of this epigenetic phenomenon in relation to DNMT2 [14]. However, the family Drosophilidae is among the most diverse of the Diptera, including more than 4,200 species [15], each with its diversified ecological, historical and evolutionary context. Species of this family, especially of the genus *Drosophila*, are widely used in many areas of contemporary biological research, and few of them have been investigated with respect to the occurrence of DNA methylation.

The distribution of DNMT2 enzymes in the Diptera reveals that DNMT2 protein sequences are highly conserved in *Drosophila virilis*, *D*. *hydei*, *D*. *simulans*, *D*. *melanogaster* and *D*. *pseudoobscura* [13]. This conservation occurs primarily within the catalytic DNA methyltransferase motifs [13]. Focusing on the *Drosophila* genus, the comparison between the DNMT2 protein sequences of *D*. *willistoni* and *D*. *melanogaster* revealed higher conservation at the domains putatively responsible for methyl transfer catalysis and great variability in the region containing the specific DNA target recognition domain (TRD) [16].

Another interesting aspect is the presence of differential cleavage patterns between males and females in the *Drosophila willistoni*, presenting a DNA methylation phenomenon that does not occur in *D*. *melanogaster* [16]. Further investigations with more species of the same *Sophophora* subgenus of *Drosophila* with respect to putative differences between sexes demonstrated that this phenomenon only occurs in sibling species of the *willistoni* subgroup [17]. The studies also established phylogenetic correlations in the sex-specific methylation patterns in the subgroup *willistoni* species, where *D*. *willistoni*, *D*. *tropicalis* and *D*. *insularis* (closer species) share methylation patterns in ribosomal genes, and the *D*. *equinoxialis* and *D*. *paulistorum* patterns are not restricted to rDNA [16,17]. Accordingly, these results suggest that selection for different targets of methylation may even occur between closely related species.

The diversity of action of DNMT2 can be verified by the findings where *Geobacter sulfurreducens* DNMT2 (*Gs*DNMT2) shows low methylation activity in tRNA-Asp and it has preferably methylates of the cytosine 28 of tRNA-Glu [18]. The trade in the specificity of *Gs*DNMT2 from tRNA-Asp to tRNA-Glu is related to changes within the *Gs*DNMT2 protein and the *Gs*-tRNAs. However, these modifications are only efficient when combined, because *Gs*DNMT2 still methylates tRNA-Asp from other species.

In addition to evolutionary studies based on molecular markers, another powerful tool that can substantially contribute to evolutionary studies is comparative-homology modelling, which is an approach that has great success for the prediction of protein 3D structures [19,20]. This process derives from some assumptions that protein structures have a higher degree of conservation than their amino acid sequences [21]. Significant progress in the prediction algorithms of the tertiary protein structure has been made in recent decades [22]. With the increased accuracy of these methods, a new dimension in the evolutionary analysis of proteins is envisioned.

Furthermore, the analysis of primary and tertiary protein sequences provides interesting suggestions regarding the evolution of protein function [23]. Clearly, the protein spatial organization tends to be more conserved than nucleotide and the amino acid sequence during evolution. However, the increasing information about three-dimensional protein structures has shown that homologous proteins might be structurally different, despite the high conservation of their primary sequence and functional similarities [24]. Tertiary structure protein analyses contribute to a better understanding of the evolution of protein functionality. From the organization's knowledge of the different domains that compose a protein, it is possible to compute molecular surfaces with an electrostatic potential. The electrostatic interactions are the fundamental driving force underlying biological processes, playing an important role in protein-ligand interaction and protein-protein molecular recognition [25].

Our objectives in present work were to analyse the structural and physicochemical characteristics evolution of the DNMT2 in Drosophilidae comparing to *Spodoptera frugiperda* [26], human [27], *Entamoeba histolytica* [28], *Haemophilus haemolyticus* [29], [30], *Haemophilus influenzae* [31] DNMT2 and *Geobacter sulfurreducens* DNMT2 [18]. For this, we perform molecular homology modelling and determination of the electrostatic potential molecular surface of the DNMT2 enzymes. The analysis from 3D structures would launch a new layer for an understanding of the variations in the function and the recognition mechanisms presented in the DNMT2 enzyme and for predicting the impact of mutations through its evolution.

## Materials and methods

### Sequences and crystallographic structures retrieving

*In silico* searches were performed to identify DNMT2 homologous sequences among the 24 sequenced *Drosophila* genomes (S2 Table) available in the FlyBase database (http://flybase.bio.indiana.edu/blast/). In most cases, the sequence annotated as “DNMT2” was directly recovered. In the other genomes, the *D*. *melanogaster* sequence (Flybase Annotation symbol: Dmel/CG10692) was used as query on BLASTn. The *D*. *willistoni* sequence used in this study was the isoform B, described by Garcia et al. (2007), which has 341 amino acids. The *D*. *buzzattii* and *D*. *suzukii* DNMT2 were obtained from the *Drosophila buzzatii* Genome Project server (http://dbuz.uab.cat/welcome.php) and SpottedWingFlyBase (http://spottedwingflybase.oregonstate.edu/), a dedicated online resource for *Drosophila suzukii* genomics. All nucleotide and amino acid sequences were then aligned using the Muscle tool [32]. The *Mus musculus* DNMT2 sequence (NP_034197) was obtained on BLASTp using as query the human DNMT2 sequence and the *Geobacter sulfurreducens* DNMT2 directly from accession number (GSU0227) from Uniprot (http://www.uniprot.org/).

Using as query sequence of *D*. *melanogaster* and human DNMT2, we performed a search through the program BLAST (Basic Local Alignment Search Tool) by homologous structures in the Protein Data Bank (PDB).

### Molecular modelling of dnmt2

The DNMT2 models were generated using a homology modelling approach. The Modeller 9.14 [33] program was applied using the *Spodoptera frugiperda* (PDB 4H0N) [26] and human DNMT2 structures as templates (PDB 1G55) [27], according to the best alignment between the template and the target sequence. We used the complete DNMT2 sequences obtained by searching *in silico*. The alignment was further verified manually and adjusted, considering the location of insertion/deletion in loops. The homology modelling was performed with a semi-automated approach, using Python scripts previously developed by our group. The modelling protocol followed the default optimization and refinement protocol, as described in the Modeller online manual (available at http://salilab.org/modeller/9.13/manual/node19.html). One hundred models were generated, and the best model was selected using the DOPE score and Procheck [34].

The generated models were evaluated by Ramachandran plot analysis, which is a well-known evaluation tool to assess the stereochemical quality of a model through the analysis of *phi* and *psi* angles for all protein residues and an overall model quality evaluation was performed using Qmean6 [35] and Verify3D [36]. The DNMT2 models were used for subsequent analysis.

### Structural conservation analyses

Sequence and structure analyses are often split into two separate approaches in evolutionary research. In this study, we conducted analyses on the structures of the DNMT2 models generated and their sequences using the Multiseq [37] program incorporated in the VMD 1.9.2 [38] as a plugin tool. The structural homology measure is based on the structural similarity measure, Q*H*, which was designed to include the effects of the gaps on the aligned portion [39].

### Electrostatic potential molecular surface analyses

For the calculation of the Poisson-Boltzmann equation, the *Adaptive Poisson-Boltzmann Solver* software (APBS) [40] was used, which is able to describe the electrostatic interactions between the molecule and solvent. The APBS was run as a plugin in 1.9 programs Chimera [41] and VMD 1.9.1.

Comparative analysis of the electrostatic properties of proteins was performed using the PIPSA algorithm (Protein Similarity Property Interaction Analysis) [42]. The program permits the classification of proteins according to the properties of molecular interactions fields, and the electrostatic potential molecular surface is the most informative molecular interaction field in these cases.

## Results

### Molecular modelling DNMT2

The search in the PDB database returned seventeen crystallographic structures of DNMT2 (S1 Table). In the present study, we used *S*. *frugiperda* [26], human [27], *E*. *histolytica* [28], *H*. *haemolyticus* [29] and *H*. *influenzae* [31] crystallographic models.

To analyze the structural conservation and emerging physicochemical properties of the tertiary structure of DNMT2 we generated twenty-six DNMT2 models of species whose genomes are deposited in databases from their amino acid residue sequences. The models of the DNMT2 protein structure were approved by Verify 3D analysis and presented a high percentage (>86%) of residues with an average 3D-1D score > 0.2. In the Ramachandran plot, all models presented around 90% of residues the most favoured region. A small percentage of residues of the models were in the disallowed regions (in the region of 1%, in every models), and most of these belonged to loop structures of the molecules (S2 Table).

The overall model evaluations were performed by Qmean6. The QMEAN6 score is composite by a linear combination of 6 terms (estimated model reliability between 0–1), with respect to scores obtained for high-resolution experimental structures of similar size solved by X-ray crystallography. The models were concluded to be of good quality, according to the z-scores obtained by Qmean6 (S2 Table). The crystallographic structure of *S*. *frugiperda* DNMT2 (PDB: 4H0N) [26] was used as a quality parameter of the models.

### Differences in amino acid composition of DNMT2

Despite to be considered an enzyme with high conservation along the various taxa, even the identity between related species is not high. Between *H*. *haemolyticus* (HhaI) and *H*. *influenzae* (HaeIII) there is a divergence of 0.7370 (S3 Table). The highest divergence is found between *M*. *musculus* and HaeIII with 0.8121. The lowest divergence values are found between *D*. *sechellia*—*D*. *simulans* (0.0116) and *D*. *persimilis*–*D*. *pseudoobscura* (0.0086). In drosophilids the highest divergence found is between *D*. *willistoni*–*D*. *biarmipes* (0.3021).

**Fig 1:**
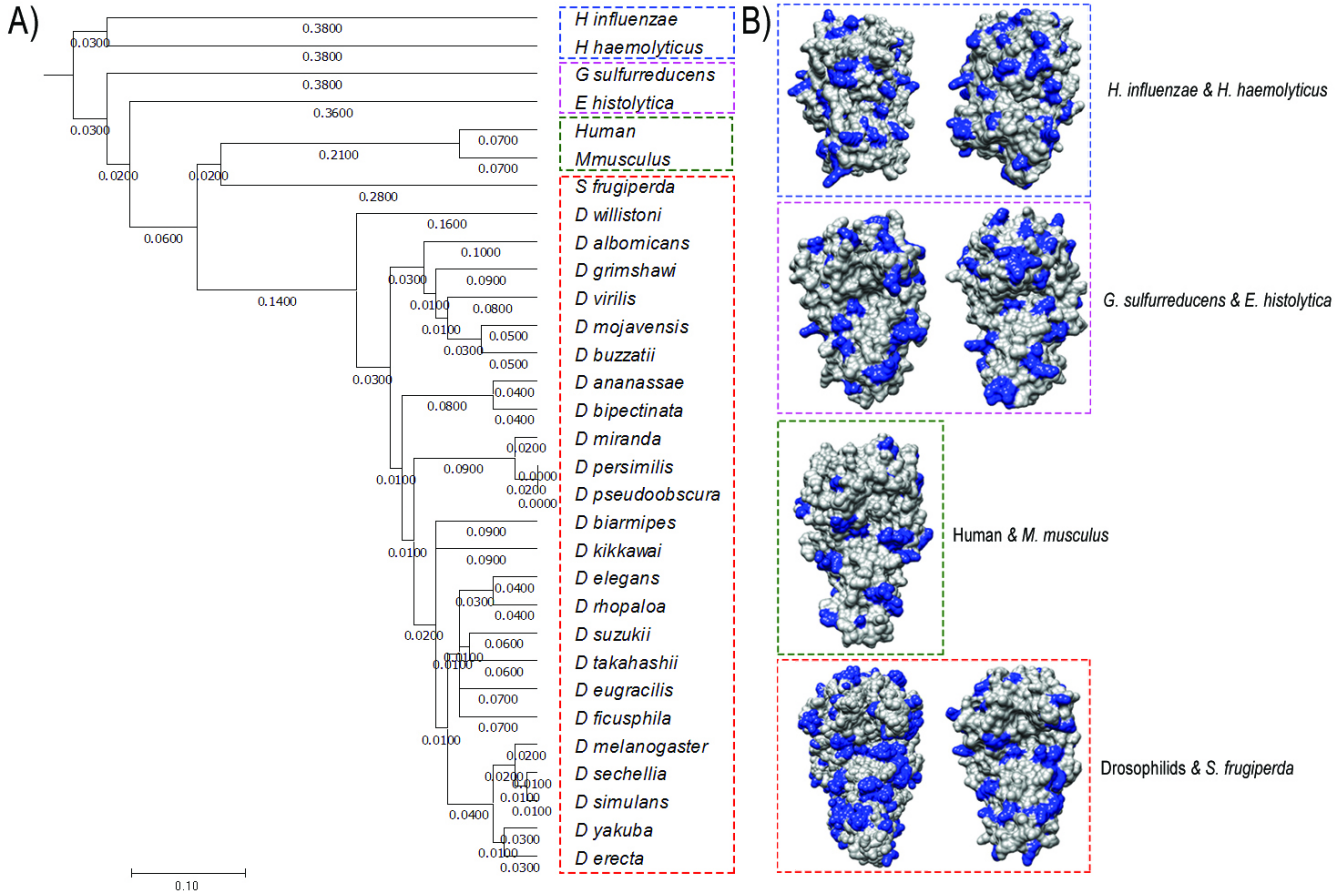
**(A)** Phylogenetic relationships based on the identity of DNMT2 sequences. The numbers above the branches are the percentage identity distance. **(B)** Representation of eukaryotes DNMT2 frontal structures with surface colored gray. All drosophilids structures are superimposed. The positively charged residues (histidine, lysine, and arginine) are colored blue.

Interestingly, *D*. *willistoni* are in a position more ancestral in the phylogenetic relationship based on identity, grouping externally to *Drosophila* subgenus (Fig 1A). *D*. *bipectinata* and *D*. *ananassae* are placed out of *melanogaster* group when was expected a basal positioning. The other evolutionary relationships between species are established as expected.

Comparatively analyzing the distribution of basic residues on the surface of eukaryotes DNMT2 we can observe a higher prevalence of basic residues in drosophilids DNMT2 in relation to vertebrate (Fig 1B). In contrast, *Gs*DNMT2 differs both as structural conformations as in the presence of basic amino acids in a different pattern from other eukaryotes DNMT2 (Fig 1B).

**Fig 2:**
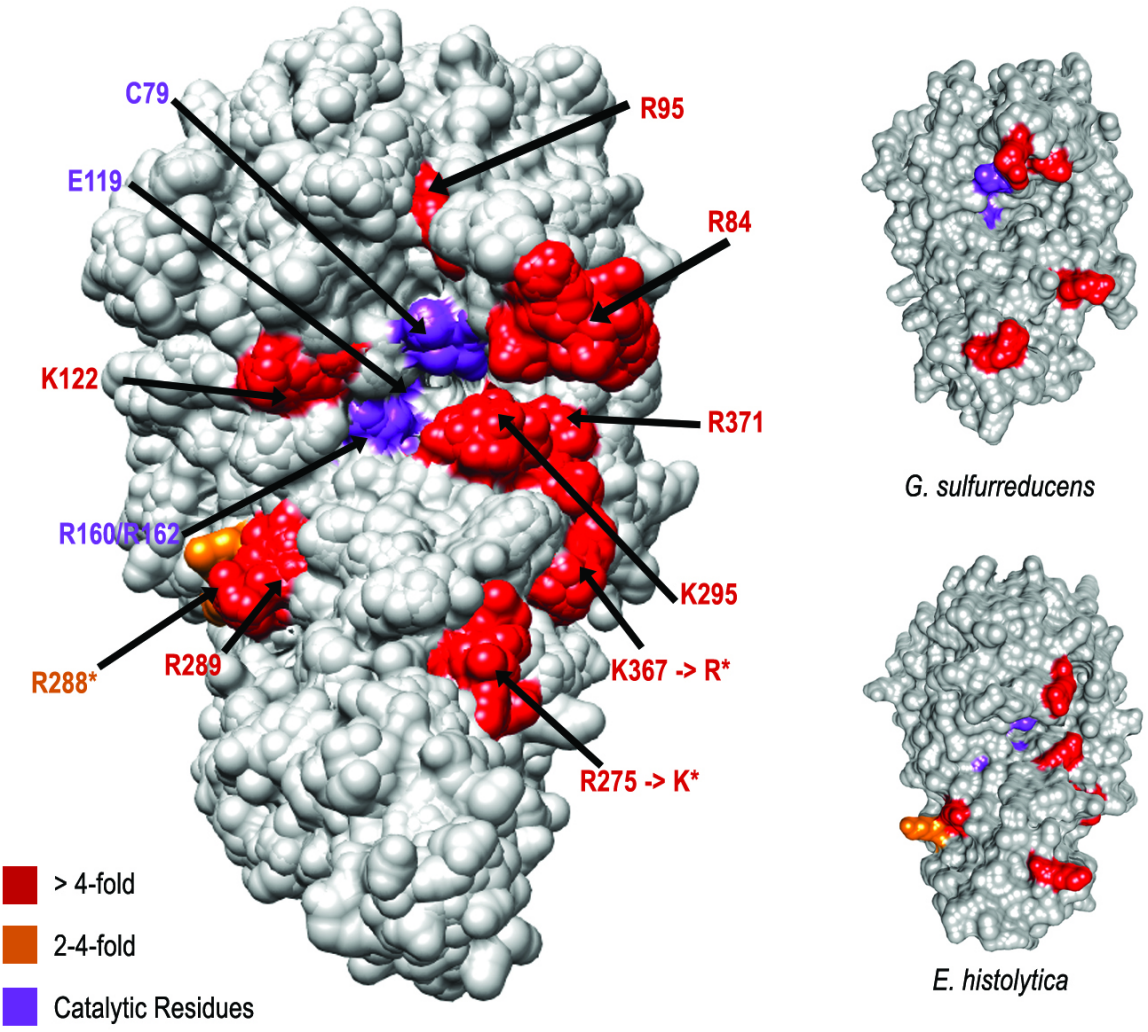
Superposition of drosophilids and human DNMT2 surface representations showing the conservation of residues that have a strong influence in the binding site, according to Jurkowski et al. 2012 where residues that strongly interfere with catalysis activity (> 4-fold) are colored red, 2-4-fold orange and catalytic residues colored purple. The residues K367 and R275 from human DNMT2 correspond to arginine (R) and lysine (K) in drosophilids, respectively. The *G*. *sulfurreducens* and *E*. *histolytica* DNMT2 surfaces are presented separately.

We assess the conservation of key residues involved in the substrate interaction according to the experimental data [43]. The residues that interfere strongly in the catalytic action of human DNMT2 are present in DNMT2 of drosophilids with synonymous mutations of physicochemical properties in two sites: R275>K and K367>R (Fig 2). However, in *Gs*DNMT2 are not present the key residues K122, R289, K295 and R371. In Ehmeth there is no match in three keys residues: R84, K122 e R371 (Fig 2).

### The DNMT2 present structural conservation of catalytic domain, but differs in TRD

**Fig 3:**
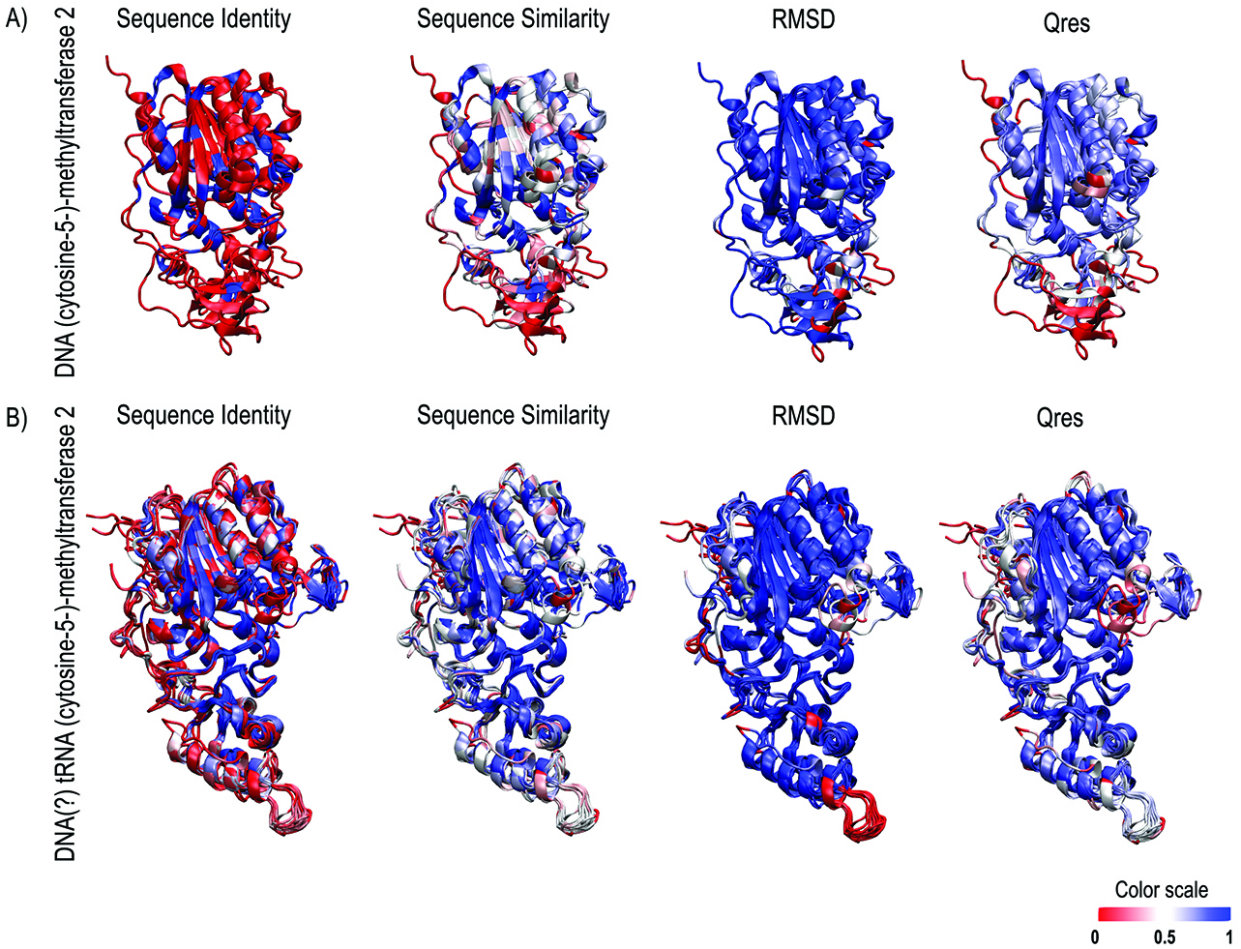
Multiple structural alignments of all DNMT2 by STAMP in VMD-multiseq. The structures are colored by sequence identity, similarity, RMSD and Qres values (where 1 indicates that the structures are identical and 0 more dissimilar). **(A)** Overriding DNMT2 of HhaI (PDB 1MHT) and HaeIII (PDB 1DCT) structures. **(B)** Superposition of remaining DNA/tRNA MTase2 structures, including *Gs*DNMT2.

The structural homology measure of the DNMT2 models was conducted by Multiseq and Chimera programs (Fig 3). The molecules were coloured according to the sequence identity, and we can measure the charge variation per site from the structures. The conserved residues are located mostly in the catalytic region, whereas the low identity residues are located in the connection of the catalytic domains and TRD. Additionally, TRD has a lower amino acid identity, despite the preservation of signature peptides in the area (motifs CFT and E/DGTS) (Fig 3B).

There is also a structural difference in the substrate recognition domain: an insertion (approximately 9 residues) in various drosophilids between the characteristic α-helix/loop/α–helix motif of this region, except in *D*. *ananassae*, *D*. *bipectinata*, *D*. *grimshawi*, *D*. *tropicalis* and *D*. *willistoni*. As expected, structural variation can be observed between closed species, as the genus *Haemophilus* (Fig 3A), since these proteins have different target-sites methylation. Throughout the evolution, the C-terminal region of TRD gradually assumed a more structured architecture with the appearance of the characteristic structural motif α-helix/loop/α-helix.

### Analyzing the differences in surface electrostatic potentials of the mutated MT2

**Fig 4:**
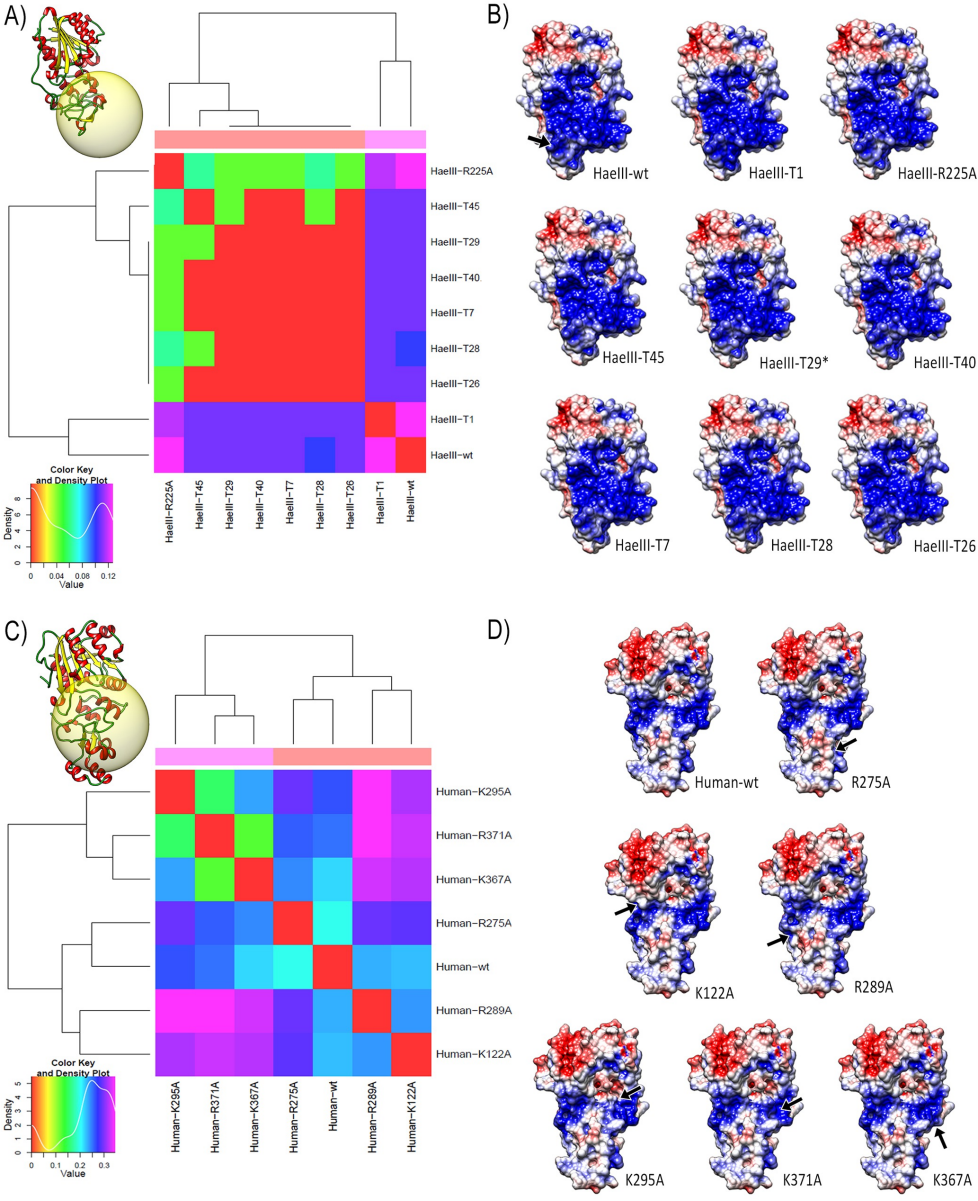
Epogram of MT2 structures. The molecules are grouped according to the similarity of the electrostatic potentials from the distance matrix constructed by PIPSA tool. The colour key, above left, indicates the relationship of similarity: where red indicates maximum similarity, whereas violet indicates low similarity. The surfaces are coloured according to the electrostatic potential: Blue for positive potential (5 kT), red for negative (-5 kT) and white for neutral, where kT = kilotesla. The black arrow in the top left structure indicates the target region of mutated residues. **(A)** Epogram showing the clusters of the comparison of HaeIII electrostatic potentials, at up left are shown the ribbon representation of the region for comparison of the electrostatic potential. **(B)** Electrostatic potential surface of HaeIII structures (substrate interface). **(C)** Human MT2 epogram from comparison of electrostatic potentials, at up left are shown the ribbon representation of the region for comparison of the electrostatic potential. **(D)** Electrostatic potential surface of human MT2 structures (substrate interface) and respective mutations.

Cohen et al. (2004) altered the sequence specificity of HaeIII by directed evolution *in vitro*. The mutant variations showed three new target sites efficiently: AGCC, CGCC and the original GGCC, which the methylation in CGCC site is slightly more efficiently than AGCC [44]. Thus we promoted the respective mutations in HaeIII, according to Cohen et al. experiment (Table 1) for the comparative analysis of the electrostatic properties (Fig 4A and 4B).

**Table 1:** Sequence of HaeIII mutants from Cohen et al. 2004.

Type	Residue				AGCC activity	GGCC activity
	225	260	261	262		
Wild-type	R	N	L	N	<0.05	50.0
R225A	A	N	L	N	0.96	1.2
T29	A	L	M	W	33.0	5.1
T7	A	L	F	W	3.9	0.12
T28	A	L	S	W	3.0	0.41
T26	A	L	T	W	2.9	0.32
T45	A	L	W	W	2.3	0.32
T1	A	L	R	W	2.0	0.10
T40	A	L	C	W	1.8	0.071

HaeIII-wt appears distantly from all other HaeIII mutated (Fig 4A). Interestingly the change of sequence specificity in HaeIII occurs by only the changing of four residues (HaeIII-T29), which impacts the electrostatic potentials surface, causing a decrease in the positive electrostatic potential in the mutated region (Fig 4B).

We also elaborate eight human DNMT2 structures with the six respective mutations that strongly interfere with catalysis activity (Table 2). In this case, the analyses of differences in surface electrostatic potentials for each mutant type appear to be more drastic in response to mutated residues (Fig 4C and 4D).

**Table 2:** Sequence of human DNMT2 mutants from Jurkowski et al. 2012.

Type	Residue	WT % activity	Deviation (%)
wt		100.00	-
Mutant-I	K295A	20.24	±1.71
Mutant-II	K122A	18.54	±6.71
Mutant-III	R289A	19.27	±9.02
Mutant-IV	R371A	14.15	±2.44
Mutant-V	K367A	10.37	±2.07
Mutant-VI	R275A	6.46	±1.71

### Extensive comparative analysis of the electrostatic properties of DNMT2 surface

As the interaction between the substrate (DNA, RNA or tRNA) occurs distributed around a region, with multiples residues interacting with ligand we posed the analysis probe in a central position in DNMT2 TRD. This position for TRD is the classical CFT motif present in eukaryotes MT2 (not present in prokaryotes, corresponding spatially to TLS and VQA motifs from HhaI and HaeIII, respectively). The region is assigned with a radius of 35Å around the correspondent TRD.

**Fig 5:**
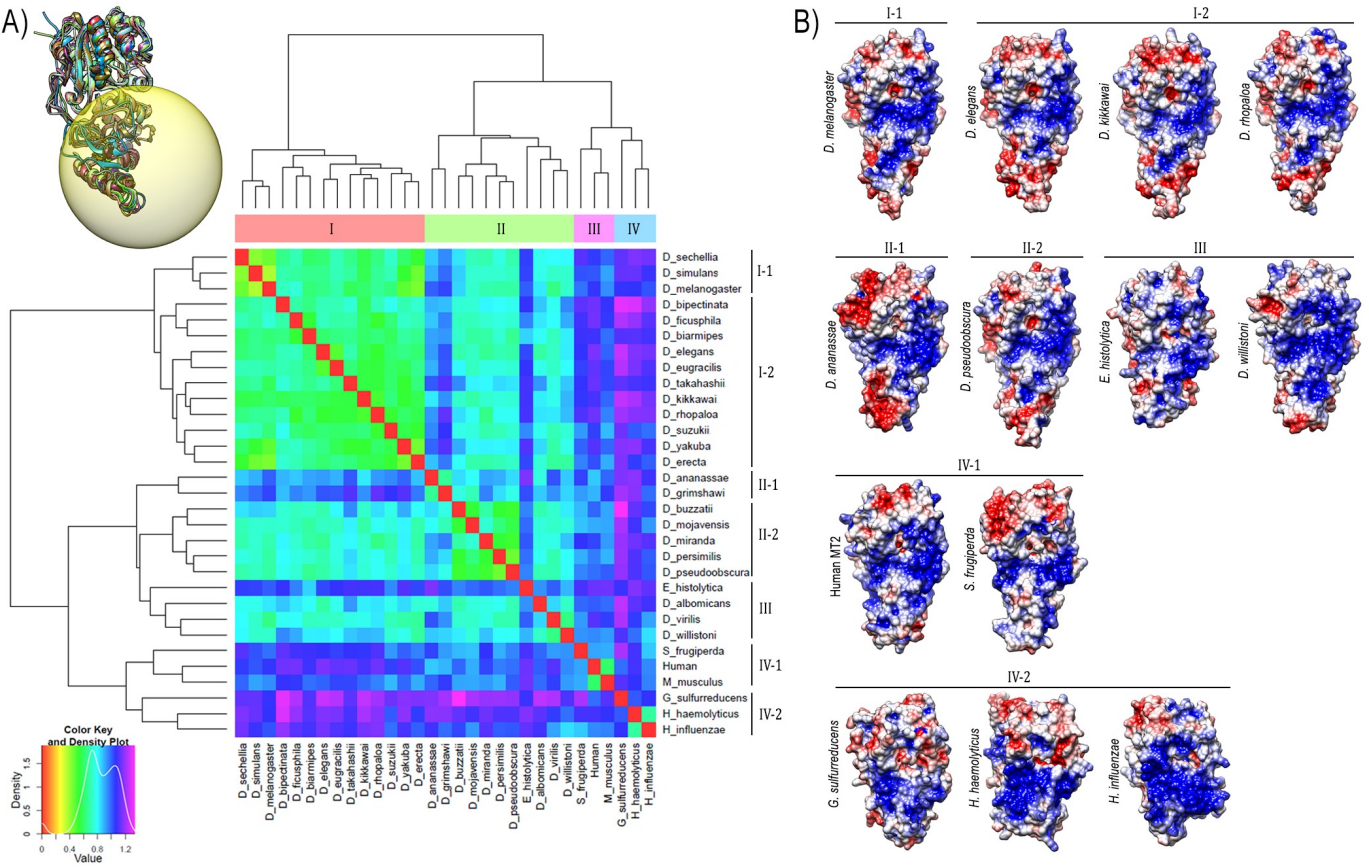
**(A)** Epogram of MT2 structures. The molecules are grouped according to the similarity of the electrostatic potentials from the distance matrix constructed by the program PIPSA. The colour key, above left, indicates the relationship of similarity: red indicates maximum similarity, whereas violet indicates low similarity. **(B)** Electrostatic potential surface of DNMT2 structures (substrate interface). The surfaces are coloured according to the electrostatic potential: blue for positive potential (5 kT), red for negative (-5 kT) and white for neutral, where kT = kilotesla. The structures are grouped according to the similarity of the distribution profile of electrostatic potentials.

The comparison of the electrostatic interaction properties of proteins analysis resulted in four main clusters: I, II, III and IV (Fig 5A). The cluster I is mostly for species of the *melanogaster* group, sharing high similarity in the electrostatic surface profile (Fig 5A and 5B). A subcluster I-1 can be found, which *D*. *sechellia*, *D*. *simulans* and *D*. *melanogaster* show the lowest electrostatic distance values: 0.3715 (*D*. *simulans*-*D*. *melanogaster*), 0.4243 (*D*. *simulans*-*D*. *sechellia*) and 0.4604 (*D*. *sechellia*-*D*. *melanogaster*) (Fig 5A). The subcluster I-2 also shares high similarity between the species and the distribution of charges on its surface (Fig 5B).

The cluster II is very heterogeneous through the similarities and distribution of charges on its surface, being composed of species from *obscura* subgroup, *Drosophila* subgenus and *D*. *ananassae* (*Sophophora* subgenus) (Fig 5A). Here we can recognize two distribution of surface electrostatic charges patterns: one represented by *D*. *ananassae* electrostatic potential surface (cluster II-1) and other represented by *D*. *pseudoobscura* pattern (cluster II-2) (Fig 5B). Clusters III and IV also behave proteins with heterogeneous surface properties. In cluster III we find the *Entamoeba histolytica*, *D*. *albomicans*, *D*. *virilis* and *D*. *willistoni* structures, however only *D*. *virilis* and *D*. *willistoni* share similarity in its electrostatic potential surfaces, with a distance value of 0.6387. The cluster IV-1 groups Human, *M*. *musculus* and *S*. *frugiperda*, but the similarity between *S*. *frugiperda-M*. *musculus* and *S*. *frugiperda-*Human MT2 are low, distance values 0.9044 and 0.9154 respectively (Fig 5A and 5B). At least, in the cluster IV-2 we find de DNA methyltransferase from HhaI and HaeIII. The *G*. *sulfurreducens* is also grouping together with HhaI and HaeIII but does not share similarities with prokaryotes DNMT2.

Interestingly, the human and *M*. *musculus* DNMT2 stand out in comparison with other MT2 molecules analyzed the smaller area of positive electrostatic potential fields in their TRD. On the other hand, the electrostatic surface of prokaryote HaeIII e HhaI is characterized by a prominent prevalence of positive charges in the TRD (Fig 5B). The lower prevalence of positive potential in the interaction interface with the substrate appears to be a feature of tRNA (cytosine-5)-methyltransferase.

The drosophilids electrostatic potential surface profile is somehow heterogeneous between them. It is clear that the distribution pattern of surface charges in drosophilids DNMT2 differs from Ehmeth and GsDNMT2, the same occurring with human and *M*. *musculus* DNMT2 (Fig 5B).

### Predicting kinetic constants K_cat_/K_m_ from DNMT2

The reaction mechanisms of DNMT2 have not been studied extensively, except for the prokaryotes HhaI and HaeIII, which have consistency measure for its kinetic constants (Table 3). Molecular electrostatic potential differences seem to correlate with kinetic rates and this correlation can be used to predict enzyme kinetic parameters [45]. The parameters K_cat_/K_m_ are linked with the region responsible for the interaction between the substrate and the enzyme, so the electrostatic potential closer to the active site presents significant for the kinetic parameter values.

**Table 3:** Kinetic constants for DNA methyltransferase from HaeIII and HhaI.

Organism	K_cat_/K_m_	K_cat_/K_m_ (ln)	Reference
*H*. *influenzae* (HaeIII)	2.60E+04	1.02E+01	[44]
*H*. *haemolyticus* (HhaI)	1.20E+07	1.63E+01	[46]

**Fig 6:**
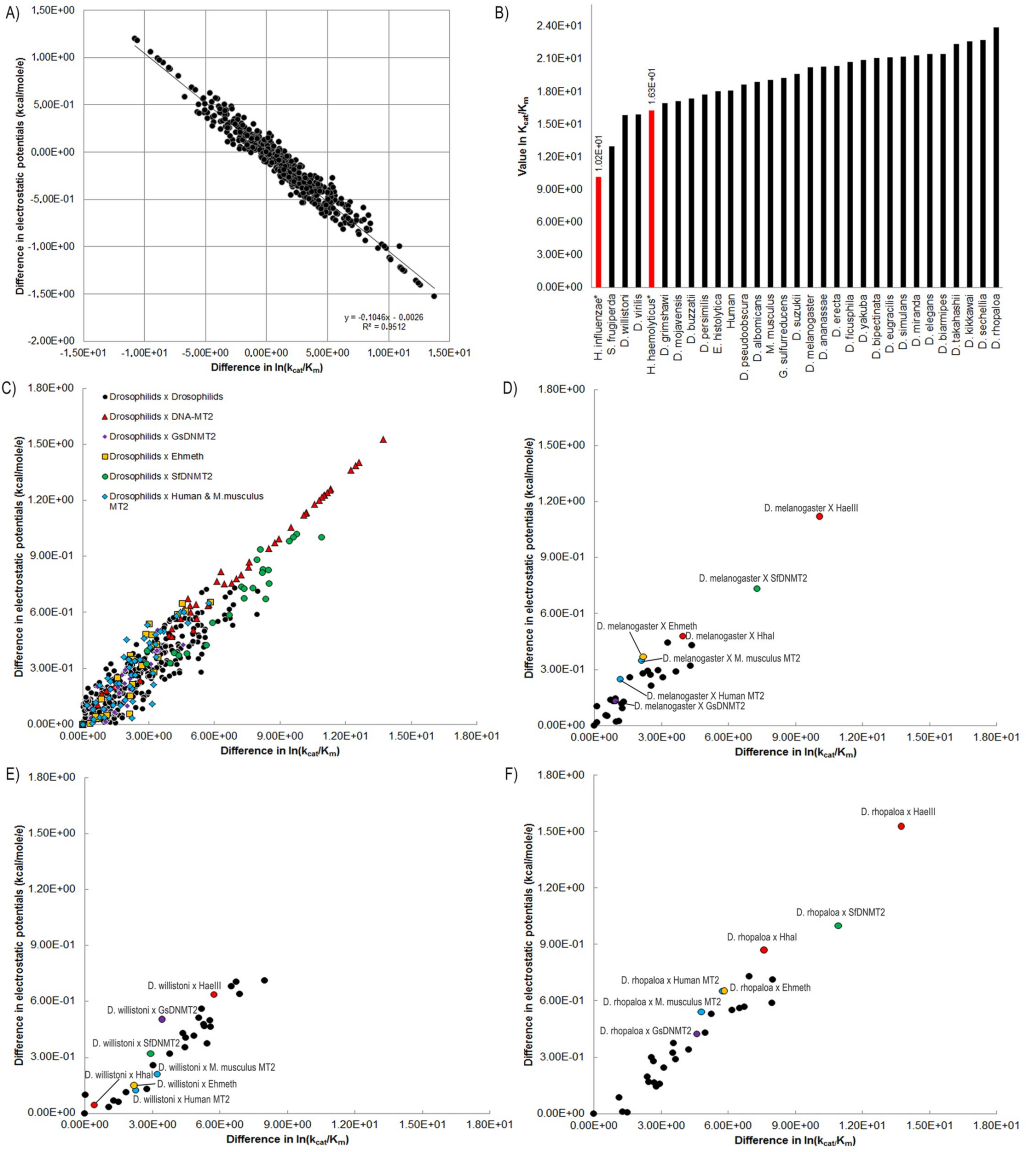
**(A)** Correlation between experimental ln K_cat_/K_m_ values and the electrostatic potential differences. **(B)** Predicted K_cat_/K_m_ from methyltransferase 2 structures. The experimental values from HhaI and HaeIII are highlighted in red. **(C)** Clusters from correlation ln K_cat_/K_m_ and electrostatic potential differences setting drosophilids correlations with all others. The values were transformed to the same quadrant. **(D)** Correlation between ln (K_cat_/K_m_) for *D*. *melanogaster* DNMT2 and electrostatic potential differences for each DNMT2 protein pair. **(E)** Correlation between ln (K_cat_/K_m_) for *D*. *willistoni* DNMT2 and electrostatic potential differences for each DNMT2 protein pair. **(F)** Correlation between ln (K_cat_/K_m_) for *D*. *rhopaloa* DNMT2 and electrostatic potential differences for each DNMT2 protein pair.

An excellent linear correlation between calculated differences in electrostatic potentials and kinetic values could be achieved (R^2^ = 0.9512) and this can be used to predict ln K_cat_/K_m_ values for the other MT2 (Fig 6A). Thus, we used the kinetic constants K_cat_/K_m_ rate from HhaI and HaeIII to infer the measure the kinetic values for the others DNMT2 analyzed in present work. For the K_cat_/K_m_ parameter, we find that an increase of K_cat_/K_m_ of 1 ln unit is related to a decrease of 0.1046 kcal/mol/e in electrostatic potential of surface residues, approximately (Fig 6A). In the extreme points, we have the differences between the pairs HaeIII-*D*. *yakuba* (-1.08E+01; 1.20E+00) and *D*. *rhopaloa*- HaeIII (1.37E+01; -1.53E+00).

HaeIII has the lowest ln K_cat_/K_m_ value of all (1.02E+01), while HhaI has ln K_cat_/K_m_ value 1.63E+01. Interestingly, *D*. *willistoni* (1.59E+01), *D*. *virilis* (1.59E+01) and *S*. *frugiperda* (1.30E+01) show a predicted ln K_cat_/K_m_ values between HaeIII and HhaI. Instead, *D*. *rhopaloa* show the highest ln K_cat_/K_m_ (2.39E+01) (Fig 6B). When we clustered the values from differences in ln K_cat_/K_m_ and electrostatic potentials, setting the drosophilids vs all other DNMT2, we can observe that the most drosophilids DNMT2 shares similarity with vertebrates DNMT2, Ehmeth and GsDNMT2, while depart from DNA-MT2 (HaeIII and HhaI) and from *Sf*DNMT2 (Fig 6C). Analyzing separately *D*. *willistoni*, *D*. *melanogaster* and *D*. *rhopaloa*, we can see more clearly the differences and similarities pair to pair with theses MT2. *D*. melanogaster seems to be more similar to the other drosophilids regarding to the differences in the electrostatic potentials surface with average differences 0.181 Kcal/mol/e (Fig 6D), while D. *willistoni* and *D*. *rhopaloa* seems to be more dissimilar with the other drosophilids MT2, averages 0.366 and 0.322 Kcal/mol/e, respectively (Fig 6E and 6F). However, *D*. *willistoni* MT2 shows greater similarity with HhaI (0.045 Kcal/mol/e) and HaeIII (0.637 Kcal/mol/e), whereas *D*. *rhopaloa* has the largest difference, 0.870 Kcal/mol/e and 1.529 Kcal/mol/e, respectively, a more similar profile to *D*. *melanogaster* in relation to these prokaryotic MT2

## Discussion

In general, the DNMT2 is considered a tRNA-ASP methyltransferase than precisely a DNA methyltransferase [2,3,47]. However, the discovery of sex-specific methylation phenomenon reestablishes the importance of maintaining a critical view of the importance and the multifunctionality of DNMT2, precisely due to the peculiarities within groups of species evolutionarily close and described as DNMT2-only.

By the end of the 90s the genome of *Drosophila melanogaster* was considered methylation-free, as described in several studies that analyzed the stages of pupa and adult Drosophila [48,49]. However, studies have been presented evidences that there would be lower levels of methylation in *Drosophila* embryos and adults. The analysis employed confirmed the existence of methylation in these organisms, but with the peculiarity of being sites for CpA, CpT, and CpC in embryos of *D*. *melanogaster*, and not CpG as is commonly found in mammalian [11]. Our research group which has focused on the study of Neotropical species (as *willistoni* subgroup) showed for the first time the phenomenon sex-specific methylation in *D*. *willistoni* genome [16]. Using the Methylation-Sensitive Restriction Endonuclease (MSRE) technique and Southern blot with specific probes, the results suggested that selection for different targets of methylation may occur between different, but closely related species [16]. In this case, species from *willistoni* subgroup seems to have a different methylation sites pattern to *D*. *melanogaster*, since the restriction sites of enzymes used are 5'-GGCC-3' and 5'-AGCT-3', respectively [16,17]. Given this scenario, in the present work, we approach the evolution of DNMT2 from structural and physical chemistry perspective in search of features that shed light on the functional plurality of the enzyme, with special attention to drosophilids.

Although there maintenance of certain catalytic motifs, generally DNMT2 cannot be described as being high conserved among different organisms (Fig 1A and S1 Table), average divergence among the studied species are 40% (S1 Table). Even between drosophilids, it finds great sequences divergence. It is interesting to note that the signatures of evolutionary processes expand beyond the analysis of synonymous substitutions and not synonymous, they are also found in the conformational "clues" of new structures that may represent functional changes of proteins. Thus, 3D similarities in protein structures may exist in the absence of sequence identity [19,50]. HaeIII and HhaI share only 30% identity, but have high structural conservation demonstrated by the RMSD values and the structural differences between HaeIII and HhaI are found in TRD (Figs 1A and 4A), which is consistent with the fact that both having the same function, however, with different target-sequence recognition [29,31].

Clearly, it is observed that the DNMT2 spatial organization tends to be more conserved than their sequence during evolution (Fig 3A and 3B), majority into the catalytic domain, as expected, since the maintenance of catalytic function in different DNMT2. However, structural differences of TRD are more pronounced when comparing the DNA-DNMT2 to tRNA-(DNA)DNMT2. The Ehmeth TRD assumes an intermediate architecture between the two DNMT2 types but has a more juxtaposed overlap with tRNA-(DNA)DNMT2. The differences both in composition and architecture of DNMT2 here studied are linked to differences in biological functions and characteristics of action of TRD on the substrate observed in the DNMT2 of different species, which even in close species (like *H*. *haemolyticus* and *H*. *influenzae*) present a high difference in composition, structure and recognition mechanisms. GsDNMT2 shows a structural composition more closed to Ehmeth than others eukaryote DNMT2, remembering that it has a preference for tRNA-Glu than the tRNA-asp substrate. The changes in the target substrate recognition are closely linked with enzyme and substrate architecture at the same time in this case [18].

Electrostatic interactions of a protein are characterized as long-range attractive forces, capable of interfering with the association rate between two molecules, as a cofactor-protein and protein-substrate and it is another interesting level to analyzing when we study the evolutionary modifications. For being a strong and long-range force, the knowledge of how the electrostatic potential is distributed on the surface of a protein ends up being critical to understanding the behavior and function of a molecule.

The alteration of sequence specificity or the processivity of an enzyme can be obtained by the changing of only one or a couple of residues, sometimes. Cohen et al. (2004) altered the target-sequence of MT2 HaeIII from GGCC to AGCC by the mutation of residues R225, N260, L261 and N262. These mutations also modified the electrostatic potentials surface, decreasing substantially the positive charge in the mutated region (Fig 4A and 4B). In another experiment, Jurkowski et al. (2012) systematically mutated lysine and arginine residues to alanine to study the methylation activity and binding of the variants and found eight residues that caused a strong decrease in catalytic activity. Despite many variants shown strong reduce of catalytic activity, only a weak loss of tRNA binding or even bound better to tRNA wild-type DNMT2. In this case, probably the DNMT2 can induce conformational changes in tRNA before the transfer reaction of a methyl group to target cytosine. As expected, the analysis of differences in surface electrostatic potentials for each mutant correlates with the experimentally observed loss of activity (Fig 4C and 4D).

Interestingly, the extensive analysis of electrostatic potential surfaces shown that the evolutionarily related groups have striking differences in how the charges are distributed on the surface of their molecules when the expectation was for drosophilids been clustering with low difference distance (Fig 5A and 5B). However, drosophilids were grouped into two main clusters, with the high distance between the species from these two clusters. The cluster-II is very heterogeneous with regard to the distribution profile of electrostatic potentials (Fig 5B). It draws attention to the electrostatic potential surface of *D*. *willistoni* in relation to other drosophilids. Visual analysis of the distribution of the *D*. *willistoni* DNMT2 surface charges reveals a larger area with a positive electrostatic profile in TRD of this Neotropical species (Fig 5B), in addition to group separately from the other species of the subgenus *Sophophora*.

For the prediction of kinetic constants from DNMT2 was taken into account that the kinetic parameters are linked with the region responsible for the interaction between the substrate and the enzyme [45]. From linear correlation calculated differences in electrostatic potentials the predicted kinetic values could be achieved (Fig 6A). The average values ln K_cat_/K_m_ for prokaryote DNMT2 are 1.32E+01 kcal/mol/e, when for drosophilids the average show higher predicted values, 2.04E+01 kcal/mol/e. However, *D*. *willistoni*, *D*. *virilis* and *S*. *frugiperda* have predicted kinetic values very close to the HaeIII and HhaI, which can suggest a differential reactivity for the same substrate of prokaryotes DNMT2, in the case, DNA (Fig 6B). It is clear the heterogeneity of drosophilids MT2 (Fig 6C–6F).

The kinetic parameter K_cat_/K_m_ measures the affinity to the transition state. Often a very reactive substrate will have a high K_m_ value since it will react faster than it is released from the enzyme unreacted. Together with the pattern of distribution of positive and negative net charges of the molecular surface, these data are in agreement with the fact that surface residues can influence kinetic values not only via the potential in the active site region but also influence the substrate affinity as it approaches the active site [45].

The differences found here may be related to the various peculiarities present in the evolutionary history of each of the studied strains. Even among drosophilids, which we would expect similarity of potentials between species, their evolutionary histories differ greatly in several aspects. For example, the ways the transpositions of TEs are regulated are mainly associated with epigenetic mechanisms [51,52]. The distribution of TEs and repetitive sequences in many *Drosophila* genomes vary greatly in relation to the percentage found: *D*. *simulans* 2.7%, *D*. *willistoni* 16% and *D*. *ananassae* 25% [53]. The events of TEs mobilization may be related to the occurrence of breaks and rearrangements of chromosomal arms, modifications to regulatory gene sequences, or even the complete inactivation of a gene, fulfilling an important role in molecular evolution of organisms, being able to promote the emergence of new genes, as well as new pathways in cellular signaling network and *D*. *willistoni* is known as a specie with high chromosomal polymorphism [54].

On the other hand, the differences between the *D*. *willistoni* and other species DNMT2 may be related to the processivity of the enzyme with the RNA substrate. Besides the genomic cytosine methylation as a process to silencing TEs, the silencing of retrotransposons and the control of RNA viruses in *Drosophila* can be mediate by DNMT2, playing an important role as a control mechanism to stress that involves RNA, like retrotransposons [55,56]. DNMT2 is an essential part of the RNA processing machinery during cellular stress [57]. In *Drosophila*, when in heat stress conditions, DICER-2 enzyme degrades tRNA and tRNA fragments, so DNMT2 then goes into action to limits the extent of tRNA fragmentation during the stress event, because too long double-stranded RNAs inhibit DICER-2 activity [56,58]. More recently, it was surprisingly found that DNMT2 methylates more efficiently DNA fragments when presented as covalent DNA-RNA hybrids in the structural context of a tRNA [59]. Such context containing both RNA and DNA covalently linked would be found in replication forks, transcription sites, DNA repair events, or retroviral replication. Moreover, it seems that the DNMT2 substrate recognition process is linked to the recognition of at least two levels: structural and primary sequence [59].

Whereas *willistoni* subgroup have sex-specific methylation of cytosine in its genome (present only in drosophilids belonging to this subgroup) [16], a high degree of chromosomal polymorphism [54,60], a wide distribution area—where many people are exposed to different environmental scenarios -, may favor a selection toward some degree of differential modulation of the DNMT2 function. *D*. *willistoni* also has a high percentage of enzyme polymorphism, around 60% [61]. Among the species of *Drosophila*, *D*. *willistoni* also shows a reduction in the diversion of use of codons—which is related to the improvement of protein synthesis—in relation to other drosophilids [62,63]. Thus, it is expected in the subgroup *willistoni* mechanisms ensuring adequate genomic stability but at the same time have sufficient plasticity to allow rapid adaptive capacity in various environments.

## Conclusions

Studies about the functional diversities of macromolecules can lead to a better understanding of the biological diversity that exists in nature. The macro-aspects (molecule as a whole and interactions with other molecules) and micro (physicochemical characteristics, distribution of electrostatic potentials, behavior in physiological environment and conservation of residues) are fundamental for the understanding of which and how different evolutionary mechanisms can be crucial in preserving or acquiring new functions of a molecule. In the present work, it was verified that DNMT2 of evolutionarily close species have different characteristics in the distribution of surface potentials, mainly in the region responsible for the recognition of the target sequence. This feature may be involved with different affinities between the enzymes for its different substrates (DNA, RNA or tRNA) and even for the epigenetic mechanisms involved throughout the evolutionary history of the species.

In the case of the Neotropical species of the *willistoni* subgroup, we suggest that there may be an intermingled system of intrinsic factors (presence of TEs and chromosomal polymorphism, for example) and extrinsic (geographic distribution and variable niches) that would be acting as evolutionary forces in the emergence and maintenance of differences observed in DNMT2. These aspects raise new questions about the importance of DNMT2 in the ontological, ecological and evolutionary aspects of this peculiar group of drosophilids.

## Supporting information

S1 Table DNMT2 crystallographic models deposited in the database Protein Data Bank used to analyze and select the template structure for homology modeling process and evolutionary studies. (DOCX)

S2 Table Percentage (%) of protein residues in each region of the Ramachandran Plot and additional evaluation tools for model viability assessment. (DOCX)

S3 Table Estimates of evolutionary divergence between sequences. The number of amino acid differences per site from between sequences are shown. Standard error estimate(s) are shown above the diagonal. The analysis involved 31 amino acid sequences. All ambiguous positions were removed for each sequence pair. There were a total of 385 positions in the final dataset. Evolutionary analyses were conducted in MEGA7.
(DOCX)
