# Supplementary material for: Linking epigenetic function to electrostatics: The DNMT2 structural model example
Source: PLoS One. 2017 Jun 2;12(6):e0178643. doi: 10.1371/journal.pone.0178643 (PMC5456315; doi:10.1371/journal.pone.0178643)
Supplement: S1 Table — (DOCX) [file pone.0178643.s001.docx]

**S1 Table.** DNMT2 crystallographic models deposited in the database Protein Data Bank used to analyze and select the template structure for homology modeling process and evolutionary studies.

| **DNMT2** | ***D. melanogaster* vs. crystal sequence Identity (%)** | ***G. sulfurreducens* vs. crystal sequence Identity (%)** | ***M. musculus* vs. crystal sequence Identity (%)** | **PDB ID** |
| --- | --- | --- | --- | --- |
| SfDNMT2 (*S. frugiperda*)* | 43.0 | 26.9 | 44.1 | 4H0N |
| Human DNMT2* | 41.0 | 35.1 | 82.8 | 1G55 |
| Ehmeth (*E. histolytica*)* | 30.0 | 24.9 | 34.6 | 3QV2 |
| HhaI (*H. haemolyticus*)* | 24.0 | 19.7 | 21.7 | 1MHT |
| HaeIII (*H. influenzae*)* | 25.0 | 22.0 | 20.4 | 1DCT |
| M.MpeI (*M. penetrans*) | 22.0 | 21.3 | 16.0 | 4DKJ |

* Structures used in the analyzes
