# Supplementary material for: Linking epigenetic function to electrostatics: The DNMT2 structural model example
Source: PLoS One. 2017 Jun 2;12(6):e0178643. doi: 10.1371/journal.pone.0178643 (PMC5456315; doi:10.1371/journal.pone.0178643)
Supplement: S2 Table — (DOCX) [file pone.0178643.s002.docx]

**S2 Table.** Percentage (%) of protein residues in each region of the Ramachandran Plot and additional evaluation tools for model viability assessment.

| Species | PROCHECK | | | |  | Qmean6 |  | Verify 3D |  |
| --- | --- | --- | --- | --- | --- | --- | --- | --- | --- |
|  | Most favored | Additional allowed | Generously allowed | Disallowed |  | Z-score |  | Residues Averaged 3D-1D > 0.2a | Results |
| *D. albomicans* | 91.8 | 7.5 | 0.3 | 0.3 |  | -1.80 |  | 87.25 | Aproved |
| *D. ananassae* | 91.1 | 7.8 | 0.7 | 0.4 |  | -1.89 |  | 92.51 | Aproved |
| *D. biarmipes* | 90.9 | 7.7 | 0.7 | 0.7 |  | -1.34 |  | 89.15 | Aproved |
| *D. bipectinata* | 86.9 | 11.3 | 0.7 | 1.1 |  | -1.93 |  | 97.02 | Aproved |
| *D. buzzatii* | 90.9 | 7.3 | 1.0 | 0.7 |  | -2.07 |  | 86.98 | Aproved |
| *D. elegans* | 90.3 | 8.3 | 1.0 | 0.3 |  | -2.10 |  | 88.01 | Aproved |
| *D. erecta* | 91.8 | 7.2 | 0.3 | 0.7 |  | -1.48 |  | 89.31 | Aproved |
| *D. eugracilis* | 91.0 | 7.6 | 1.0 | 0.3 |  | -1.33 |  | 89.47 | Aproved |
| *D. ficusphila* | 90.6 | 8.4 | 0.7 | 0.3 |  | -1.83 |  | 89.11 | Aproved |
| *D. grimshawi* | 90.3 | 8.3 | 0.0 | 1.4 |  | -1.79 |  | 91.76 | Aproved |
| *D. kikkawai* | 89.5 | 9.1 | 1.0 | 0.3 |  | -2.07 |  | 93.33 | Aproved |
| *D. melanogaster* | 90.6 | 8.7 | 0.0 | 0.7 |  | -1.39 |  | 91.01 | Aproved |
| *D. miranda* | 89.1 | 9.2 | 1.0 | 0.7 |  | -1.84 |  | 90.43 | Aproved |
| *D. mojavensis* | 91.7 | 7.6 | 0.3 | 0.3 |  | -2.01 |  | 89.57 | Aproved |
| *D. persimilis* | 90.2 | 8.1 | 1.0 | 0.7 |  | -1.71 |  | 90.20 | Aproved |
| *D. pseudoobscura* | 90.5 | 7.8 | 1.0 | 0.7 |  | -1.67 |  | 93.08 | Aproved |
| *D. rhopaloa* | 89.5 | 9.2 | 1.0 | 0.3 |  | -2.00 |  | 94.51 | Aproved |
| *D. sechellia* | 90.7 | 7.6 | 1.0 | 0.7 |  | -2.01 |  | 90.70 | Aproved |
| *D. simulans* | 91.0 | 7.6 | 0.7 | 0.7 |  | -1.26 |  | 89.28 | Aproved |
| *D. suzukii* | 90.2 | 7.8 | 1.0 | 1.0 |  | -1.71 |  | 93.73 | Aproved |
| *D. takahashii* | 90.7 | 7.6 | 1.0 | 0.7 |  | -1.93 |  | 92.44 | Aproved |
| *D. virilis* | 90.0 | 9.0 | 0.7 | 0.3 |  | -2.09 |  | 92.20 | Aproved |
| *D. willistoni* | 88.6 | 10.7 | 0.7 | 0.0 |  | -1.89 |  | 90.32 | Aproved |
| *D. yakuba* | 91.8 | 7.2 | 0.3 | 0.7 |  | -1.20 |  | 89.28 | Aproved |
| *M. musculus* | 92.0 | 6.6 | 1.0 | 0.3 |  | -0.37 |  | 92.54 | Aproved |
| *G. sulfurreducens* | 90.2 | 8.6 | 0.8 | 0.4 |  | -1.20 |  | 92.79 | Aproved |
| *S. frugiperda* (4H0N) | 91.5 | 8.5 | 0.0 | 0.0 |  | -1.06 |  | 98.19 | Aproved |
| ^a^ Values given in %; | | | | | | | | | |
