# Supplementary material for: Linking epigenetic function to electrostatics: The DNMT2 structural model example
Source: PLoS One. 2017 Jun 2;12(6):e0178643. doi: 10.1371/journal.pone.0178643 (PMC5456315; doi:10.1371/journal.pone.0178643)
Supplement: S3 Table — The number of amino acid differences per site from between sequences are shown. Standard error estimate(s) are shown above the diagonal. The analysis involved 31 amino acid sequences. All ambiguous positions were removed for each sequence pair. There were a total of 385 positions in the final dataset. Evolutionary analyses were conducted in MEGA7. (DOCX) [file pone.0178643.s003.docx]

**S3 Table.** Estimates of Evolutionary Divergence between Sequences. The number of amino acid differences per site from between sequences are shown. Standard error estimate(s) are shown above the diagonal. The analysis involved 31 amino acid sequences. All ambiguous positions were removed for each sequence pair. There were a total of 385 positions in the final dataset. Evolutionary analyses were conducted in MEGA7.

|  |  | 1 | 2 | 3 | 4 | 5 | 6 | 7 | 8 | 9 | 10 | 11 | 12 | 13 | 14 | 15 | 16 | 17 | 18 | 19 | 20 | 21 | 22 | 23 | 24 | 25 | 26 | 27 | 28 | 29 | 30 | 31 |
| --- | --- | --- | --- | --- | --- | --- | --- | --- | --- | --- | --- | --- | --- | --- | --- | --- | --- | --- | --- | --- | --- | --- | --- | --- | --- | --- | --- | --- | --- | --- | --- | --- |
| 1 | S_frugiperda |  | 0.0264 | 0.0261 | 0.0261 | 0.0258 | 0.0269 | 0.0258 | 0.0256 | 0.0255 | 0.0254 | 0.0257 | 0.0258 | 0.0257 | 0.0260 | 0.0265 | 0.0259 | 0.0259 | 0.0259 | 0.0258 | 0.0258 | 0.0256 | 0.0259 | 0.0262 | 0.0258 | 0.0255 | 0.0262 | 0.0262 | 0.0254 | 0.0247 | 0.0227 | 0.0215 |
| 2 | D_albomicans | 0.5640 |  | 0.0225 | 0.0223 | 0.0224 | 0.0204 | 0.0218 | 0.0213 | 0.0213 | 0.0226 | 0.0215 | 0.0214 | 0.0215 | 0.0221 | 0.0201 | 0.0222 | 0.0222 | 0.0213 | 0.0212 | 0.0211 | 0.0210 | 0.0211 | 0.0197 | 0.0234 | 0.0210 | 0.0263 | 0.0266 | 0.0256 | 0.0259 | 0.0223 | 0.0214 |
| 3 | D_ananassae | 0.5485 | 0.2424 |  | 0.0219 | 0.0145 | 0.0224 | 0.0206 | 0.0211 | 0.0209 | 0.0199 | 0.0233 | 0.0204 | 0.0215 | 0.0220 | 0.0225 | 0.0218 | 0.0218 | 0.0207 | 0.0212 | 0.0213 | 0.0208 | 0.0206 | 0.0222 | 0.0234 | 0.0204 | 0.0265 | 0.0267 | 0.0252 | 0.0255 | 0.0222 | 0.0212 |
| 4 | D_biarmipes | 0.5675 | 0.2544 | 0.2287 |  | 0.0223 | 0.0219 | 0.0185 | 0.0187 | 0.0195 | 0.0193 | 0.0219 | 0.0206 | 0.0198 | 0.0214 | 0.0221 | 0.0213 | 0.0210 | 0.0194 | 0.0192 | 0.0192 | 0.0161 | 0.0182 | 0.0216 | 0.0245 | 0.0200 | 0.0260 | 0.0264 | 0.0258 | 0.0261 | 0.0225 | 0.0223 |
| 5 | D_bipectinata | 0.5576 | 0.2424 | 0.0838 | 0.2287 |  | 0.0219 | 0.0212 | 0.0218 | 0.0218 | 0.0211 | 0.0230 | 0.0213 | 0.0222 | 0.0221 | 0.0225 | 0.0218 | 0.0218 | 0.0215 | 0.0221 | 0.0219 | 0.0213 | 0.0215 | 0.0218 | 0.0236 | 0.0210 | 0.0265 | 0.0267 | 0.0249 | 0.0254 | 0.0228 | 0.0217 |
| 6 | D_buzzatii | 0.5590 | 0.1982 | 0.2407 | 0.2418 | 0.2438 |  | 0.0216 | 0.0222 | 0.0216 | 0.0224 | 0.0197 | 0.0221 | 0.0223 | 0.0223 | 0.0148 | 0.0223 | 0.0222 | 0.0218 | 0.0222 | 0.0222 | 0.0219 | 0.0214 | 0.0175 | 0.0234 | 0.0218 | 0.0261 | 0.0266 | 0.0252 | 0.0263 | 0.0228 | 0.0225 |
| 7 | D_elegans | 0.5798 | 0.2278 | 0.1890 | 0.1525 | 0.1890 | 0.2269 |  | 0.0185 | 0.0181 | 0.0182 | 0.0213 | 0.0195 | 0.0183 | 0.0206 | 0.0217 | 0.0206 | 0.0204 | 0.0143 | 0.0180 | 0.0183 | 0.0167 | 0.0171 | 0.0211 | 0.0251 | 0.0182 | 0.0260 | 0.0266 | 0.0256 | 0.0261 | 0.0226 | 0.0213 |
| 8 | D_erecta | 0.5680 | 0.2176 | 0.2096 | 0.1593 | 0.2066 | 0.2432 | 0.1559 |  | 0.0178 | 0.0193 | 0.0213 | 0.0196 | 0.0139 | 0.0207 | 0.0226 | 0.0208 | 0.0207 | 0.0179 | 0.0131 | 0.0125 | 0.0186 | 0.0173 | 0.0218 | 0.0242 | 0.0124 | 0.0262 | 0.0266 | 0.0252 | 0.0257 | 0.0224 | 0.0214 |
| 9 | D_eugracilis | 0.5818 | 0.2189 | 0.2018 | 0.1543 | 0.2078 | 0.2296 | 0.1331 | 0.1374 |  | 0.0176 | 0.0215 | 0.0192 | 0.0190 | 0.0200 | 0.0217 | 0.0200 | 0.0200 | 0.0171 | 0.0184 | 0.0186 | 0.0172 | 0.0175 | 0.0214 | 0.0243 | 0.0178 | 0.0257 | 0.0260 | 0.0250 | 0.0260 | 0.0221 | 0.0213 |
| 10 | D_ficusphila | 0.5727 | 0.2471 | 0.1862 | 0.1598 | 0.2000 | 0.2523 | 0.1445 | 0.1536 | 0.1144 |  | 0.0228 | 0.0190 | 0.0198 | 0.0204 | 0.0225 | 0.0210 | 0.0208 | 0.0177 | 0.0195 | 0.0196 | 0.0179 | 0.0168 | 0.0216 | 0.0232 | 0.0188 | 0.0257 | 0.0261 | 0.0250 | 0.0256 | 0.0220 | 0.0210 |
| 11 | D_grimshawi | 0.5518 | 0.1953 | 0.2576 | 0.2380 | 0.2606 | 0.1717 | 0.2139 | 0.2209 | 0.2132 | 0.2478 |  | 0.0213 | 0.0217 | 0.0213 | 0.0196 | 0.0219 | 0.0217 | 0.0217 | 0.0217 | 0.0217 | 0.0216 | 0.0214 | 0.0191 | 0.0227 | 0.0212 | 0.0261 | 0.0266 | 0.0255 | 0.0263 | 0.0222 | 0.0218 |
| 12 | D_kikkawai | 0.5576 | 0.2141 | 0.1928 | 0.1888 | 0.1958 | 0.2515 | 0.1676 | 0.1628 | 0.1637 | 0.1545 | 0.2186 |  | 0.0199 | 0.0208 | 0.0214 | 0.0208 | 0.0206 | 0.0189 | 0.0198 | 0.0196 | 0.0193 | 0.0184 | 0.0212 | 0.0234 | 0.0192 | 0.0263 | 0.0268 | 0.0250 | 0.0259 | 0.0219 | 0.0214 |
| 13 | D_melanogaster | 0.5788 | 0.2206 | 0.2132 | 0.1805 | 0.2162 | 0.2372 | 0.1504 | 0.0754 | 0.1496 | 0.1681 | 0.2239 | 0.1633 |  | 0.0203 | 0.0228 | 0.0208 | 0.0207 | 0.0181 | 0.0109 | 0.0094 | 0.0180 | 0.0169 | 0.0214 | 0.0241 | 0.0148 | 0.0262 | 0.0267 | 0.0251 | 0.0261 | 0.0221 | 0.0213 |
| 14 | D_miranda | 0.5671 | 0.2522 | 0.2152 | 0.2324 | 0.2152 | 0.2567 | 0.2000 | 0.1935 | 0.1858 | 0.1912 | 0.2156 | 0.2076 | 0.2029 |  | 0.0227 | 0.0081 | 0.0077 | 0.0206 | 0.0204 | 0.0203 | 0.0205 | 0.0206 | 0.0220 | 0.0228 | 0.0204 | 0.0257 | 0.0258 | 0.0257 | 0.0260 | 0.0218 | 0.0213 |
| 15 | D_mojavensis | 0.5532 | 0.1977 | 0.2387 | 0.2493 | 0.2477 | 0.0888 | 0.2255 | 0.2500 | 0.2315 | 0.2529 | 0.1652 | 0.2353 | 0.2412 | 0.2559 |  | 0.0227 | 0.0226 | 0.0213 | 0.0224 | 0.0225 | 0.0220 | 0.0217 | 0.0179 | 0.0234 | 0.0222 | 0.0256 | 0.0260 | 0.0247 | 0.0257 | 0.0227 | 0.0222 |
| 16 | D_persimilis | 0.5719 | 0.2463 | 0.2091 | 0.2255 | 0.2108 | 0.2485 | 0.1899 | 0.1994 | 0.1840 | 0.1983 | 0.2232 | 0.2000 | 0.2023 | 0.0263 | 0.2522 |  | 0.0047 | 0.0207 | 0.0206 | 0.0206 | 0.0205 | 0.0205 | 0.0221 | 0.0228 | 0.0209 | 0.0259 | 0.0260 | 0.0256 | 0.0258 | 0.0216 | 0.0211 |
| 17 | D_pseudoobscura | 0.5719 | 0.2434 | 0.2091 | 0.2196 | 0.2108 | 0.2425 | 0.1869 | 0.1965 | 0.1840 | 0.1953 | 0.2173 | 0.1971 | 0.1994 | 0.0234 | 0.2463 | 0.0086 |  | 0.0204 | 0.0205 | 0.0205 | 0.0203 | 0.0204 | 0.0219 | 0.0227 | 0.0208 | 0.0259 | 0.0260 | 0.0256 | 0.0258 | 0.0216 | 0.0210 |
| 18 | D_rhopaloa | 0.5697 | 0.2222 | 0.1928 | 0.1642 | 0.1988 | 0.2239 | 0.0789 | 0.1424 | 0.1257 | 0.1283 | 0.2083 | 0.1570 | 0.1429 | 0.1953 | 0.2141 | 0.1877 | 0.1818 |  | 0.0181 | 0.0180 | 0.0174 | 0.0155 | 0.0203 | 0.0239 | 0.0174 | 0.0262 | 0.0268 | 0.0253 | 0.0256 | 0.0224 | 0.0213 |
| 19 | D_sechellia | 0.5667 | 0.2206 | 0.2078 | 0.1686 | 0.2048 | 0.2402 | 0.1475 | 0.0669 | 0.1466 | 0.1628 | 0.2239 | 0.1603 | 0.0436 | 0.2000 | 0.2441 | 0.1971 | 0.1941 | 0.1429 |  | 0.0057 | 0.0173 | 0.0167 | 0.0210 | 0.0241 | 0.0139 | 0.0262 | 0.0267 | 0.0252 | 0.0261 | 0.0227 | 0.0217 |
| 20 | D_simulans | 0.5667 | 0.2147 | 0.2072 | 0.1686 | 0.2042 | 0.2342 | 0.1475 | 0.0609 | 0.1437 | 0.1623 | 0.2209 | 0.1574 | 0.0319 | 0.2000 | 0.2382 | 0.1994 | 0.1965 | 0.1370 | 0.0116 |  | 0.0173 | 0.0167 | 0.0210 | 0.0241 | 0.0132 | 0.0261 | 0.0265 | 0.0252 | 0.0260 | 0.0222 | 0.0214 |
| 21 | D_suzukii | 0.5710 | 0.2339 | 0.2096 | 0.1056 | 0.2096 | 0.2358 | 0.1345 | 0.1503 | 0.1257 | 0.1391 | 0.2374 | 0.1715 | 0.1478 | 0.2006 | 0.2485 | 0.1977 | 0.1919 | 0.1301 | 0.1395 | 0.1391 |  | 0.0156 | 0.0210 | 0.0243 | 0.0188 | 0.0257 | 0.0262 | 0.0249 | 0.0257 | 0.0222 | 0.0211 |
| 22 | D_takahashii | 0.5727 | 0.2183 | 0.1982 | 0.1331 | 0.2042 | 0.2259 | 0.1124 | 0.1250 | 0.1235 | 0.1108 | 0.2216 | 0.1433 | 0.1195 | 0.1912 | 0.2360 | 0.1882 | 0.1853 | 0.1023 | 0.1199 | 0.1166 | 0.1017 |  | 0.0207 | 0.0238 | 0.0171 | 0.0260 | 0.0263 | 0.0253 | 0.0257 | 0.0220 | 0.0216 |
| 23 | D_virilis | 0.5714 | 0.1628 | 0.2349 | 0.2285 | 0.2380 | 0.1361 | 0.2136 | 0.2287 | 0.2107 | 0.2346 | 0.1416 | 0.2206 | 0.2141 | 0.2324 | 0.1420 | 0.2310 | 0.2251 | 0.1965 | 0.2088 | 0.2053 | 0.2274 | 0.2059 |  | 0.0234 | 0.0212 | 0.0259 | 0.0264 | 0.0253 | 0.0264 | 0.0220 | 0.0222 |
| 24 | D_willistoni | 0.5758 | 0.2651 | 0.2779 | 0.3021 | 0.2810 | 0.2615 | 0.2779 | 0.2708 | 0.2866 | 0.2567 | 0.2500 | 0.2687 | 0.2776 | 0.2613 | 0.2590 | 0.2590 | 0.2530 | 0.2687 | 0.2836 | 0.2776 | 0.2976 | 0.2716 | 0.2681 |  | 0.0239 | 0.0255 | 0.0259 | 0.0251 | 0.0258 | 0.0217 | 0.0217 |
| 25 | D_yakuba | 0.5636 | 0.2235 | 0.1892 | 0.1805 | 0.1922 | 0.2342 | 0.1534 | 0.0667 | 0.1378 | 0.1478 | 0.2299 | 0.1633 | 0.0870 | 0.2029 | 0.2412 | 0.2023 | 0.1994 | 0.1341 | 0.0756 | 0.0696 | 0.1536 | 0.1283 | 0.2258 | 0.2776 |  | 0.0262 | 0.0268 | 0.0252 | 0.0254 | 0.0221 | 0.0211 |
| 26 | Human | 0.5460 | 0.5767 | 0.5745 | 0.5710 | 0.5745 | 0.5781 | 0.5802 | 0.5897 | 0.5793 | 0.5732 | 0.5706 | 0.5854 | 0.5793 | 0.5736 | 0.5810 | 0.5692 | 0.5692 | 0.5884 | 0.5793 | 0.5823 | 0.5836 | 0.5793 | 0.5810 | 0.5775 | 0.5854 |  | 0.0181 | 0.0258 | 0.0268 | 0.0243 | 0.0216 |
| 27 | Mmusculus | 0.5446 | 0.5569 | 0.5684 | 0.5573 | 0.5636 | 0.5611 | 0.5604 | 0.5775 | 0.5688 | 0.5623 | 0.5477 | 0.5627 | 0.5701 | 0.5600 | 0.5613 | 0.5583 | 0.5583 | 0.5719 | 0.5657 | 0.5701 | 0.5684 | 0.5640 | 0.5566 | 0.5701 | 0.5732 | 0.1411 |  | 0.0256 | 0.0262 | 0.0242 | 0.0210 |
| 28 | E_histolytica | 0.6612 | 0.6743 | 0.6786 | 0.6755 | 0.6806 | 0.6846 | 0.6733 | 0.6828 | 0.6938 | 0.6817 | 0.6809 | 0.6938 | 0.6851 | 0.6743 | 0.6852 | 0.6765 | 0.6765 | 0.6840 | 0.6808 | 0.6851 | 0.6871 | 0.6786 | 0.6830 | 0.6775 | 0.6818 | 0.6634 | 0.6516 |  | 0.0236 | 0.0211 | 0.0216 |
| 29 | G_sulfurreducens | 0.7241 | 0.6817 | 0.7201 | 0.6934 | 0.7153 | 0.6961 | 0.6934 | 0.6928 | 0.6873 | 0.6959 | 0.6920 | 0.6873 | 0.6884 | 0.6955 | 0.7000 | 0.6976 | 0.6976 | 0.6976 | 0.6873 | 0.6884 | 0.7065 | 0.6962 | 0.6701 | 0.7123 | 0.7021 | 0.6542 | 0.6453 | 0.7559 |  | 0.0220 | 0.0233 |
| 30 | H_haemolyticus | 0.7822 | 0.7987 | 0.7850 | 0.7940 | 0.7702 | 0.7879 | 0.7907 | 0.8013 | 0.7934 | 0.7968 | 0.7954 | 0.8066 | 0.8072 | 0.8053 | 0.7829 | 0.8033 | 0.8033 | 0.7902 | 0.7902 | 0.8007 | 0.7980 | 0.8046 | 0.7967 | 0.8046 | 0.7941 | 0.7500 | 0.7670 | 0.8034 | 0.8000 |  | 0.0250 |
| 31 | H_influenzae | 0.7853 | 0.7987 | 0.8000 | 0.7864 | 0.7943 | 0.7883 | 0.8026 | 0.7968 | 0.7987 | 0.8063 | 0.7866 | 0.7955 | 0.8025 | 0.7942 | 0.7898 | 0.7949 | 0.7981 | 0.7987 | 0.7955 | 0.7994 | 0.8032 | 0.7937 | 0.7810 | 0.8000 | 0.8057 | 0.8051 | 0.8121 | 0.8116 | 0.7986 | 0.7370 |  |
